# Solidly Mounted Longitudinally Excited Shear Wave Resonator (YBAR) Based on Lithium Niobate Thin-Film

**DOI:** 10.3390/mi12091039

**Published:** 2021-08-29

**Authors:** Zhen-Hui Qin, Shu-Mao Wu, Yong Wang, Kang-Fu Liu, Tao Wu, Si-Yuan Yu, Yan-Feng Chen

**Affiliations:** 1 National Laboratory of Solid State Microstructures, Department of Materials Science and Engineering, Nanjing University, Nanjing 210093, China; qinzhenhui@smail.nju.edu.cn (Z.-H.Q.); wushumao2014@sina.com (S.-M.W.); 2 State Key Lab of Crystal Materials, Shandong University, Jinan 250100, China; y_wang23@163.com; 3 School of Information Science and Technology, ShanghaiTech University, Shanghai 201210, China; liukf@shanghaitech.edu.cn (K.-F.L.); wutao@shanghaitech.edu.cn (T.W.); 4 Jiangsu Key Laboratory of Artificial Functional Materials, Nanjing University, Nanjing 210093, China

**Keywords:** YBAR, bulk acoustic resonator (BAR), solid mounted resonator (SMR), LiNbO_3_ film

## Abstract

This paper proposed a solid-mounted (SM) longitudinally excited shear wave resonator (i.e., YBAR). By adopting a 200 nm x-cut LiNbO_3_ film, top (aluminum) and bottom (platinum) electrodes in 50 nm thickness and 500 nm width, this resonator simultaneously achieves an operating frequency over 5 GHz with an electromechanical coupling coefficient exceeding 50%. Compared with previously proposed YBAR with suspended structure, the proposed SM-YBAR can effectively suppress unwanted spurious modes with only a slight loss of the electromechanical coupling coefficient. The SM-YABR also provides better device stability, possible low-temperature drift coefficient, and a more convenient and mature device processing. It has the potential to meet the multiple requirements for the next generation signal processing devices in terms of high frequency, large bandwidth, stability, and low cost, etc.

## 1. Introduction

Microwave acoustic devices based on surface acoustic waves (SAW) and bulk acoustic waves (BAW) are of great value in the communication and sensing fields. The core of these devices is the microwave acoustic resonator, which can further construct today’s mainstream microwave acoustic filters, duplexers, multiplexers, and various kinds of sensors [1,2,3,4]. With the advent of the new generation of mobile communication technology (5G) and the Internet of things (IOT), the demand for high-performance microwave acoustic resonators continues to increase. At present, they are developing in the direction of having, to some extent and at the same time, a higher frequency, higher quality factor (Q), larger electromechanical coupling coefficient, smaller volume, and lower temperature coefficient (TCF) [5,6,7,8], etc.

Bulk acoustic resonators (BARs) based on single-crystal lithium niobate (LiNbO_3_) films, thanks to their ability to simultaneously meet high working frequency, large electromechanical coupling coefficient, and high Q at the same time [9,10], have recently shown a high practical application value. One of the representative BARs is the so-called XBAR [11], in which laterally-excited standing shear waves are the main resonance mode, i.e., the antisymmetric modes, in the LiNbO_3_ film. It can achieve an electromechanical coupling coefficient of more than 25% while having a working frequency over 4 GHz, further realizing a high-quality filter with a bandwidth up to hundreds of MHz [12].

**Figure 1:**
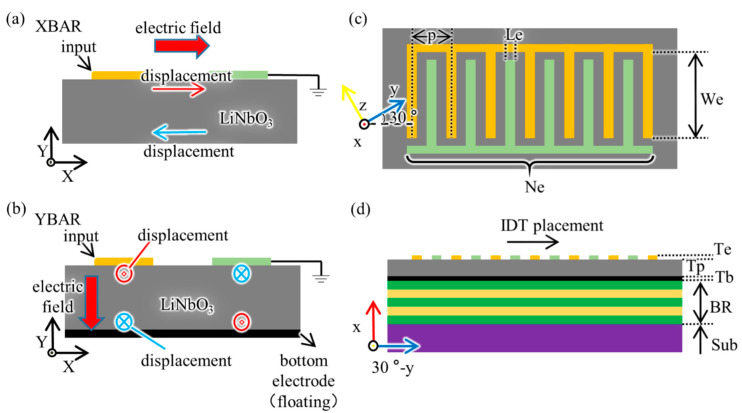
(**Left**) device principal and acoustic displacement of an XBAR and a YBAR. (**a**) an XBAR, electric field applied by the interdigital transducers (IDTs) in the transverse direction (i.e., X direction) was utilized to excite the transverse acoustic shear wave. (**b**) a YBAR, electric field applied by the IDTs in the longitudinal direction (i.e., Y direction) was utilized to excite the longitudinal acoustic shear wave. The red/blue arrows represent the displacement of the excited shear waves. (**Right**) Device configuration of the SM-YBAR in (**c**) top view and (**d**) section view, colored coordinates show the LiNbO_3_ orientation in the resonator.

Similar to the XBAR, the YBAR was proposed in 2020 [13], in which longitudinally-excited shear waves resonate in the LiNbO_3_ film. In terms of device configuration, the XBAR consists of the piezoelectric film (most likely LiNbO_3_) and interdigital electrodes (IDTs) on its top surface, while the YBAR has one more electrode at the bottom of the piezoelectric film than the XBAR. Figure 1a,b shows the differences in device configuration and acoustic resonance principle between the XBAR and YBAR. Regardless of the XBAR or YBAR, their resonance frequency mainly depends on the thickness of the piezoelectric film. Due to the existence of the bottom electrode, the resonance frequency of the YBAR is slightly lower than the XBAR at the same piezoelectric film (with the same thickness). The advantage of the YBAR is that the presence of the bottom electrode utilizes the electric field more efficiently, thus further improving the electromechanical coupling coefficient of the resonator.

In principle, for BARs based on piezoelectric films, including the XBAR and YBAR, there are two methods to suppress the radiation of the BAW from the film piezoelectric to the substrate. One is to suspend the piezoelectric film (i.e., acoustic isolation from air or vacuum), and the other is the acoustic Bragg reflector [14,15]. The Bragg reflector is composed of alternating high and low acoustic impedance layers, which can effectively reflect the downward radiated BAW in the piezoelectric film.

In this paper, we studied and proposed a solidly mounted longitudinally excited shear wave resonator (SM-YBAR) based on x-cut LiNbO_3_ film with excellent performance. In the simulation of 3D finite element analysis (FEA) software COMSOL Multiphysics, by adopting a 200 nm x-cut LiNbO_3_ film (available in the current industry), the SM-YBAR achieved a resonant frequency of over 5 GHz and a large electromechanical coupling coefficient of more than 50%. Figure 1c,d shows the structure of the proposed SM-YBAR. Compared with the previous YBAR with a suspended structure, although the electromechanical coupling coefficient of the SM-YBAR is slightly reduced, it has a significant advantage in spurious mode suppression, which means that the design of this type of YBAR will be more flexible and convenient. Additionally, the preparation of the SM-YBAR is easier to implement precisely and with lower cost, resulting from completely avoiding the etching/processing of LiNbO_3_. Moreover, devices with non-suspended structures have better mechanical stability than those that are suspended, so the SM-YABR is more suitable to meet the application requirements of various mobile scenarios in the new generation wireless communications. Below, we will discuss specifically the SM-YBAR in its design principle, material selection, and geometrical configuration with corresponding numerical calculations/simulations.

## 2. Crystal Cut and Orientation of the LiNbO_3_ Film

Due to the anisotropy of the single LiNbO_3_ [16], different crystal cut will have decisive influence on the BAWRs. For LiNbO_3_-based BAWRs (including the YABR), the x-cut LiNbO_3_ has a higher piezoelectric coefficient than other crystal cuts [17]. Consequently, we chose the x-cut LiNbO_3_ film to ensure that the YBAR has a larger electromechanical coupling coefficient.

**Figure 2:**
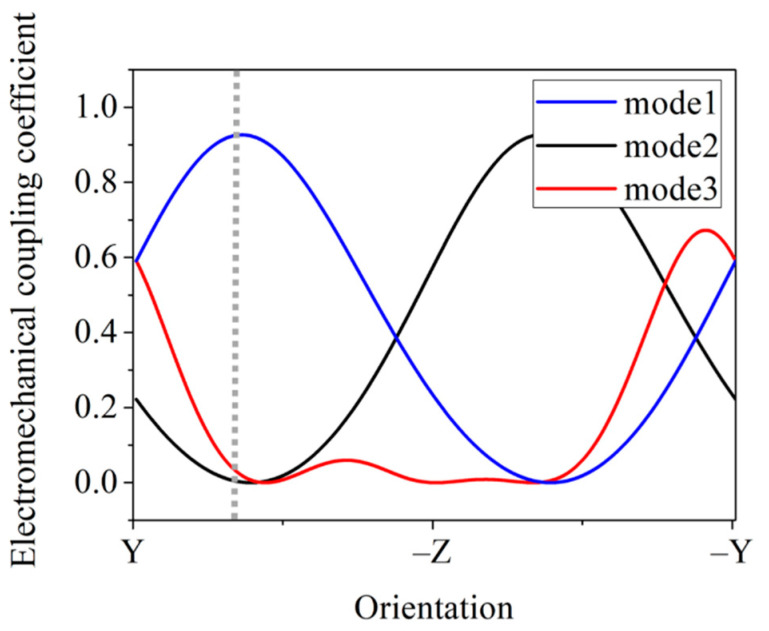
Relationship between the electromechanical coupling coefficient of an x-cut LiNbO_3_ film and the orientation of IDTs placed on its top surface.

Specifically, for the YABR, it is not only the crystal cut of LiNbO_3_ film that determines the piezoelectric coefficient but also the direction along which the IDTs are placed on the top surface of the LiNbO_3_ film. In this paper, we denoted the placement direction of IDTs as the vertical direction of their electrodes, as shown in Figure 1d, and studied how this direction affects the coupling coefficient of the x-cut LiNbO_3_ film, as shown in Figure 2. The placement is rotated clockwise from y to −y. The electromechanical coupling coefficient is determined by the formula [18]

(1) $$K_{ij}^{2}=\frac{e_{ij}^{2}}{\varepsilon_{ii}\;c_{jj}}$$

where *e_ij_* is the piezoelectric coefficient, which is the main factor determining *K^2^*, *ε_ii_* is the relative permittivity, and *c_jj_* is the elastic modulus. In Figure 2, mode1 is the main excitation mode of the YBAR with corresponding piezoelectric coefficient *e*_34_, while mode2 and mode3 are both spurious modes with corresponding piezoelectric coefficients *e*_16_ and *e*_35_, respectively. The electromechanical coupling coefficient reaches its maximum when the IDTs are placed in the direction of 30°-y, while some other spurious modes are relatively weak. Therefore, in this paper, we chose to place IDTs on the x-cut LiNbO_3_ surface along the 30°-y direction.

## 3. Thickness of the LiNbO_3_ Film

**Figure 3:**
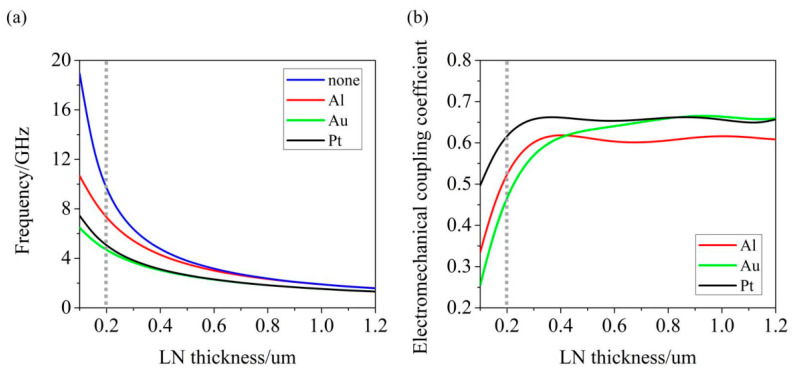
(**a**) Relationship between the thickness of the x-cut LiNbO_3_ film and the resonance frequency of the YBAR with different bottom electrode materials. (**b**) Relationship between the thickness of the x-cut LiNbO_3_ film and the electromechanical coupling coefficient of the YBAR with different bottom electrode materials.

In the YBAR, since the excited shear wave will propagate perpendicular to the piezoelectric LiNbO_3_ film, the main factor determining the resonance frequency of the YBAR is the thickness of the LiNbO_3_ film but not the period of IDTs. At the same time, the metal electrode layers above and below the LiNbO_3_ film will also have an impact on the longitudinally excited shear wave. In order to reduce this effect, the thickness of the metal electrode should be reduced as much as possible. As the calculation results show in Figure 3, without considering the metal electrode, the resonance frequency of the YBAR will increase with the decrease in x-cut LiNbO_3_ film thickness. When the film thickness is 200 nm, the resonance frequency is over 8 GHz. Considering the influence of metal electrodes (in this paper, we chose Al as the top electrode and Al, Pt, and Au as the bottom electrode, sharing the same thickness of 50 nm, considering that the electrodes should be as thin as possible and processing limitations), the resonance frequency of the YBAR will be lower, but still higher than 4 GHz. We also studied the influence of the LiNbO_3_ film’s thickness on the electromechanical coupling coefficient. When the LiNbO_3_ film is thinned to a certain thickness (around 200~300 nm), the electromechanical coupling coefficient will rapidly decrease as the film thickness decreases. When the LiNbO_3_ film thickness is 200 nm, using Pt as the bottom electrode will have a larger electromechanical coupling coefficient but a lower resonance frequency than using Al, and will have a larger electromechanical coupling coefficient and a higher resonance frequency than using Au. Hence, in the research later in this paper, we chose to adopt 50 nm Al and Pt as the top and bottom electrodes of the resonator based on 200 nm x-cut LiNbO_3_. In this choice, the YBAR would get a resonance frequency over 5 GHz and an electromechanical coupling coefficient over 60%. However, it should be noted that an Al bottom electrode is also an advantageous choice.

## 4. YBAR in Different Structures

**Figure 4:**
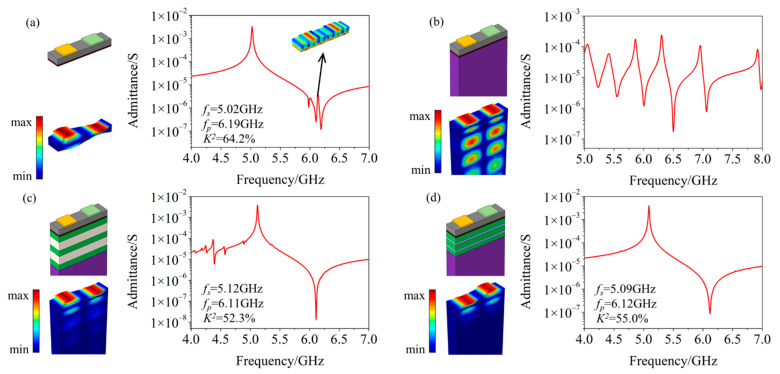
Structures, calculated admittance, and BAW field distribution in the resonance of four different YBARs. (**a**) Suspended Al-LiNbO_3_-Pt, (**b**) Al-LiNbO_3_-Pt mounted on Al_2_O_3_ substrate, (**c**) an alternately arranged SiO_2_-Al_2_O_3_ multilayer (served as a BAW Bragg Reflector) added between the Al-LiNbO_3_-Pt layer and the Al_2_O_3_ substrate, (**d**) an alternately arranged SiO_2_-Pt multilayer (also served as a BAW Bragg Reflector) added between the Al-LiNbO_3_-Pt layer and the Al_2_O_3_ substrate.

In this section, we numerically studied the performance of four kinds of YBARs (including devices A, B, C, and D) in different structures by the finite element method (FEM), using the piezoelectric module of commercial software COMSOL Multiphysics. As shown in Figure 4, in all these four devices, the thickness of the LiNbO_3_ film is set to 200 nm; the thickness of the aluminum (Al) IDTs on the top surface of the LiNbO_3_ film is 50 nm, with identical electrode width of 0.5 μm and a period of 2 μm; the thickness of the platinum (Pt) electrode at the bottom of the LiNbO_3_ film is 50 nm.

Device A is a YBAR with a suspended structure. Devices B, C, and D are SM-YBARs, all on a sapphire (Al_2_O_3_) substrate. In device B, there is only the Al_2_O_3_ substrate supporting the (from the top surface to the bottom) Al-LiNbO_3_-Pt layer. In device C, there is an alternately arranged SiO_2_-Al_2_O_3_ multilayer (i.e., SiO_2_-Al_2_O_3_-SiO_2_-Al_2_O_3_-SiO_2_) added between the Al-LiNbO_3_-Pt layer and the Al_2_O_3_ substrate. The structure of device D is similar to that of device C, while the multilayer is made of SiO_2_ and Pt (i.e., SiO_2_-Pt-SiO_2_-Pt- SiO_2_). These two kinds of alternately arranged multilayers play the role of Bragg reflectors in the BAWR, reducing the BAW radiation from the LiNbO_3_ film to the Al_2_O_3_ substrate.

In the Bragg reflectors, the Pt layers and the Al_2_O_3_ layers are high-impedance layers, while the SiO_2_ layers are low acoustic impedance layers. The greater the difference in acoustic impedance between the two adjacent layers, the better the down-warding acoustic waves will be reflected and localized inside the LiNbO_3_ film. When the thickness of each layer in the Bragg reflector is selected as λ/4 (λ is the wavelength of the acoustic wave in each layer), the Bragg reflector will have the best working ability for acoustic wave reflection [19]. Under this design, we set the thickness of the Pt, SiO_2_, and Al_2_O_3_ layers as 0.060 μm, 0.174 μm, and 0.295 μm, respectively.

In our simulations, the material parameters (elastic, piezoelectric, and dielectric constants) setting of the single crystal LiNbO_3_ film refer to the literature [1,6], and the settings of deposited SiO_2_ and Al_2_O_3_ films refer to the literature [20,21]. In addition, all losses (whether mechanical or dielectric material losses or air damping) are not considered.

Calculated BAW field displacements and admittance of the four devices are also shown in Figure 4. Additionally, according to the admittance calculation results of the four devices, we could further determine the electromechanical coupling coefficient of the resonator by the formula [22]

(2) $$K^{2}=\frac{\pi^{2}}{8}\left(\frac{f_{p}^{2}-f_{s}^{2}}{f_{s}^{2}}\right)$$

where *f_s_* is the peak value of the admittance map, and *f_p_* is the valley value. It can be seen that, except for device B, the other three devices can achieve good BAW resonance effects, and their electromechanical coupling coefficients are all over 50% (64.2%, 52.3%, and 55.0% for devices A, C, and D, respectively).

Comparing the results of device A and devices C/D, the former has obvious spurious modes near *f_p_*. Examining the BAW field displacements of these spurious modes (shown in the insets), we found that the appearance of these spurious modes is closely related to the free-boundary bottom Pt electrode of the device. In devices C and D, since the bottom Pt electrode no longer serves as a free boundary, but is sandwiched solidly between the LiNbO_3_ film and the substrate, these spurious modes are effectively suppressed.

Comparing the results of devices C and D, we found that the Bragg reflectors of different materials have little inference on resonance frequency and electromechanical coupling coefficient of the YBAR. Therefore, the materials used for the BAW Bragg reflector can be selected in various ways according to the practical device processing, the needed temperature drift of the device, and other considerations.

## 5. 3D simulation of the SM-YBAR

**Figure 5:**
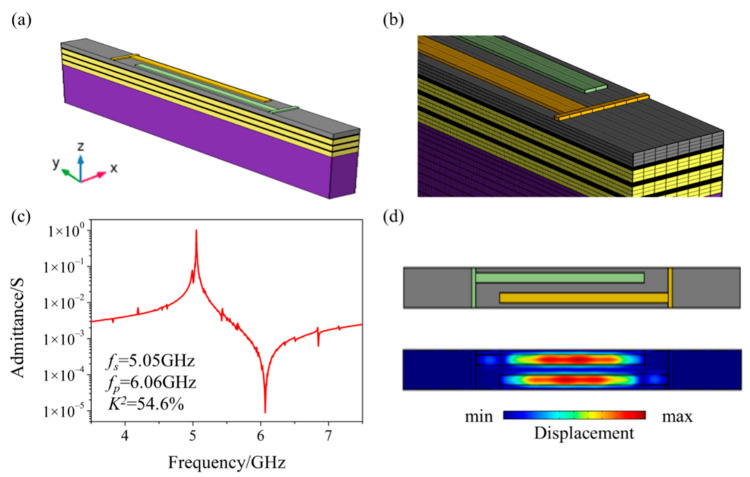
3D simulation of the SM-YBAR. (**a**) 3D schematic diagram; (**b**) Finite element meshing; (**c**) calculated admittance; (**d**) Top view and the BAW field distribution at its resonance frequency.

According to the design of device D in the previous section, we also performed a full 3D simulation of the SM-YBAR. In this simulation, both the aperture of the IDTs (set to 36 μm) and their busbar electrode are considered. Figure 5a shows the 3D model of the SM-YBAR. The boundaries in the x and y directions of the model are set as periodic boundaries and low reflection boundaries, respectively. Figure 5b shows the finite element meshing of the 3D model. The maximum size of each mesh is less than one-eighth of the IDTs’ period, small enough according to a mesh refinement study. Figure 5c,d shows its calculated admittance and the BAW field distribution at its resonance frequency.

It can be seen that the BAW at resonance is mainly concentrated in the IDTs area. This is conducive to achieving a high-quality factor (Q) of the resonator. In this paper, the simulated SM-YBAR has a theoretical Q reaching 10^4^. In practical terms, limited by the finite size of the device and all of its losses, the Q value will be lower than the simulation result. Compared with the result of the SM-YBAR obtained by the simulation in the previous section, the electromechanical coupling coefficient of the device obtained by the 3D simulation is still over 50%. There are some peaks of spurious modes in the admittance spectrum, but they are negligible compared with the main peak (the longitudinally excited shear wave), thus, hardly affecting the performance of the device.

## 6. Performance of the SM-YBAR Depending on Different IDTs Design

**Figure 6:**
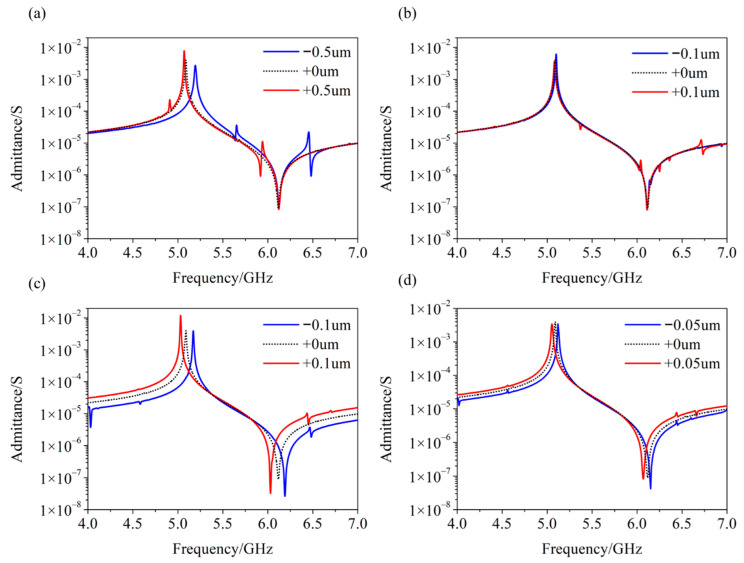
Calculated admittance of the SM-YABR depending on different period (*p*) and electrode-width (Le) of the interdigital transducers (IDTs). (**a**) *p* = 2 ± 0.5 μm, Le = 500 nm; (**b**) *p* = 2 ± 0.1 μm, Le = 500 nm; (**c**) *p* = 2 μm, Le = 500 ± 100 nm; (**d**) *p* = 2 μm, Le = 500 ± 50 nm.

Above, we have clarified the design of the SM-YBAR in the following aspects: (1) crystal cut and orientation of the LiNbO_3_ film, (2) thickness of the LiNbO_3_ film, (3) thickness and materials of the top and bottom electrodes, and (4) thickness and materials of the multilayers Bragg reflector. After these, since the SM-YABR has a relatively simple structure, there is not much room for further design, except the period and electrode-width of the IDTs. Although the electrode period does not directly determine the operating frequency of the YABR, as in SAW devices, a reasonable period helps suppress the spurious mode in the YABR. After research, it was found that when a quarter period of the IDTs was similar to the equivalent wavelength of the BAW at its working frequency, i.e., a 2 μm period of IDTs at the working frequency around 5 GHz, all spurious modes in the working bandwidth were near-perfectly suppressed, as the calculated results in Figure 6 show. Under this setting, if the electrode-width of the IDTs is changed, the working frequency of the YABR will be slightly shifted, but it will hardly affect the electromechanical coupling coefficient and suppression of the spurious modes.

## 7. A Possible Manufacturing Process for the SM-YBAR

**Figure 7:**
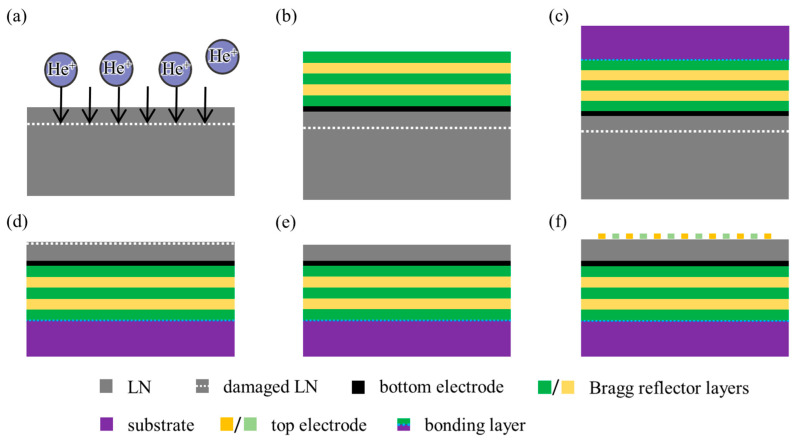
A possible manufacturing process for the SM-YBAR. (**a**) He+ (or H+) implantation in LiNbO_3_; (**b**) Bottom electrode (Pt or Al) and Bragg reflector (SiO_2_, Al_2_O_3_ or Pt) multilayers deposition; (**c**) Bonding to a substrate. The substrate material used in the article is Al_2_O_3_. It can have many choices according to the convenience of bonding and will not affect the device’s performance. (**d**) Thermal annealing to split the LiNbO_3_ film; (**e**) Polishing; (**f**) Top electrode (Al) deposition and lithographic processing to obtain the IDTs.

For our SM-YBAR, we propose a possible manufacturing process, as shown in Figure 7. Similar processes have been experimentally verified in the literature [23,24]. The 200 nm LiNbO_3_ single crystal film on the multilayer substrate can be prepared by the smart-cut technology used in many advancing BAWRs. This proposed process avoids the direct bonding of the LiNbO_3_ film to the bottom electrode. Instead, the bottom electrode is produced by depositing on the surface of the He+ implanted LiNbO_3_.

## 8. Impact of the Fabrication Process on the Performance of the SM-YABR

Due to errors in the actual manufacturing process, the actual geometric dimensions of each part of the device will inevitably deviate from the designed size to a certain extent. The geometric parameters that may deviate mainly include (1) thickness of the LiNbO_3_ film, (2) thickness of the top and bottom electrodes, (3) thickness of the multilayers in the Bragg reflector and (4) electrode-width of the IDTs.

**Figure 8:**
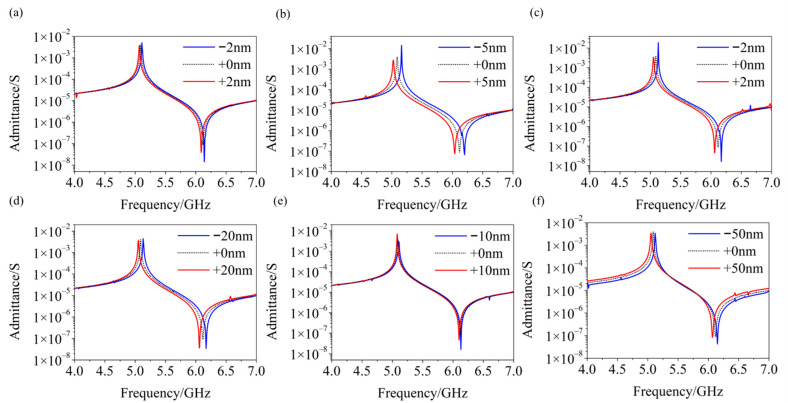
Performance of the SM-YBAR after several main geometric parameters deviate from their design values. (**a**) The thickness of the top electrode (Al, 50 ± 2 nm); (**b**) Thickness of the LiNbO_3_ film (200 ± 5 nm); (**c**) Thickness of the bottom electrode (Pt, 50 ± 2 nm); (**d**) Thickness of the SiO_2_ layers in the Bragg reflector (174 ± 20 nm); (**e**) Thickness of the Pt layers in the Bragg reflector (60 ± 10 nm); (**f**) Electrode-width of the IDTs (500 ± 50 nm).

We have investigated the device performance after these geometric parameters deviate from the design value, and the results are shown in Figure 8. Taking into account (1) different processing techniques for different components of the device, and (2) technical capabilities and production efficiency of the current industry, these deviations are set from ±2 nm to ±50 nm. It can be seen that after these major processing errors are considered, the proposed SM-YBAR still has a good performance. These errors may slightly affect the YBAR’s resonant frequency but hardly affect its electromechanical coupling coefficient, and will not introduce spurious modes into its working frequency band.

## 9. Conclusions

For the realization of a microwave acoustic resonator with high frequency (greater than 5 GHz), large electromechanical coupling coefficient (larger than 50%), high device stability, and simple preparation processing, this paper proposed a solid mounted longitudinally excited shear wave resonator (i.e., SM-YBAR) on an x-cut LiNbO_3_ film. Based on 3D simulations using the finite element method, by adopting a 200 nm LiNbO_3_ film and electrodes width of 500 nm, the SM-YBAR offers a resonance frequency exceeding 5 GHz and an electromechanical coupling coefficient exceeding 50%. Compared with the previous YBAR with a suspended structure, an SM-YABR naturally has a better stability. Additionally, its processing is more convenient for avoiding the etching of LiNbO_3_, which is still a challenge in the industry today. Even advantageously, an SM-YBAR effectively avoids the spurious modes that are prone to appear in the suspended YABR. With the rapid development of LiNbO_3_ thin-film technologies, we believe that the proposed SM-YBAR is promising for advancing high-throughput radio frequency devices in mobile communications.

## Data Availability

Not applicable.
